# Choice of Magnetometers and Gradiometers after Signal Space Separation

**DOI:** 10.3390/s17122926

**Published:** 2017-12-16

**Authors:** Pilar Garcés, David López-Sanz, Fernando Maestú, Ernesto Pereda

**Affiliations:** 1 Laboratory of Cognitive and Computational Neuroscience (UCM-UPM), Centre for Biomedical Technology, 28223 Madrid, Spain; david.lopez@ctb.upm.es (D.L.-S.); fernando.maestu@ctb.upm.es (F.M.); 2 Biomedical Research Networking Center in Bioengineering Biomaterials and Nanomedicine (CIBER-BBN), Av. Monforte de Lemos 3-5, 28029 Madrid, Spain; 3 Department of Basic Psychology II, Faculty of Psychology, Universidad Complutense de Madrid, 28223 Madrid, Spain; 4 Department of Industrial Engineering, Instituto Universitario de Neurociencia, Universidad de La Laguna, 38205 Tenerife, Spain; eperdepa@ull.edu.es

**Keywords:** magnetoencephalography, signal space separation, magnetometer, gradiometer, beamforming, regularization

## Abstract

Background: Modern Elekta Neuromag MEG devices include 102 sensor triplets containing one magnetometer and two planar gradiometers. The first processing step is often a signal space separation (SSS), which provides a powerful noise reduction. A question commonly raised by researchers and reviewers relates to which data should be employed in analyses: (1) magnetometers only, (2) gradiometers only, (3) magnetometers and gradiometers together. The MEG community is currently divided with regard to the proper answer. Methods: First, we provide theoretical evidence that both gradiometers and magnetometers result from the backprojection of the same SSS components. Then, we compare resting state and task-related sensor and source estimations from magnetometers and gradiometers in real MEG recordings before and after SSS. Results: SSS introduced a strong increase in the similarity between source time series derived from magnetometers and gradiometers (r^2^ = 0.3–0.8 before SSS and r^2^ > 0.80 after SSS). After SSS, resting state power spectrum and functional connectivity, as well as visual evoked responses, derived from both magnetometers and gradiometers were highly similar (Intraclass Correlation Coefficient > 0.8, r^2^ > 0.8). Conclusions: After SSS, magnetometer and gradiometer data are estimated from a single set of SSS components (usually ≤ 80). Equivalent results can be obtained with both sensor types in typical MEG experiments.

## 1. Introduction

The signal space separation method (SSS) [1] and its spatiotemporal extension (tSSS) [2] are powerful noise-reduction methods commonly used as a first preprocessing step in raw MEG data analysis. In fact, they have repeatedly been proven to be successful in the suppression of unwanted magnetic noise originating from distant [3] and nearby [2] sources, or even from orthodontic material [4]. Additionally, the use of device-independent coordinates in SSS enables to compensate for head movements inside the MEG scanner [2]. Roughly, this is achieved by considering the raw MEG data as a superposition of harmonic components, which originate either inside or outside the brain (or rather, a sphere that is fitted individually and lies inside the MEG helmet). SSS discards the external components and produces a cleaner version of the MEG data by backprojecting the internal components exclusively. 

SSS is a popular technique for denoising MEG data. It was recently made publicly available in MNE python for application to all whole-head MEG systems [5]. However, the use of SSS is particularly widespread in modern Elekta Neuromag^®^, Helsinki, Finland (Vectorview and TRIUX) MEG systems, since it can be easily applied directly at Elekta MEG workstations with Maxfilter^TM^ [6] or with MNE software (from version 0.11 onwards [7]). These new systems are equipped with 306 sensors, grouped into 102 elements with one magnetometer and two orthogonal planar gradiometers each [8]. Magnetometers measure the component of the magnetic field perpendicular to the MEG helmet surface (or rather, to the sensor’s coil): $B_{z}\left(\vec{r}\right)$ and are sensitive to fields originating within a wide distance. Planar gradiometers, instead, estimate the spatial derivative of $B_{z}\left(\vec{r}\right)$ in two orthogonal directions perpendicular to the MEG helmet (i.e., $\frac{\partial B_{z}\left(\vec{r}\right)}{\partial x}$ and $\frac{\partial B_{z}\left(\vec{r}\right)}{\partial y}$), so that their sensitivity decreases faster with distance. Hence, they are less sensitive to distant sources and more robust to environmental interference [9,10]. Both magnetometers and gradiometers measure tiny magnetic fields, typically in the range of 20 fT and 5 fT/cm, respectively.

Although the availability of these two sensor types at each location is very appealing, it also creates some controversy with regard to the appropriate analysis pipeline. This is particularly the case when performing source reconstruction. Researchers in the field typically follow one of three different approaches for this purpose: (1) using gradiometers only, (2) using magnetometers only, or (3) using a combination of both sensor types, usually after applying some scaling factor that yields a similar variance in the time series of both data types. Notably, some recent methodological approaches, which can deal with several sensor types and rank deficient data, enable the use of the third option. For instance, factor analysis is a useful technique for robustly estimating covariance matrices with heteroscedastic noise [11], and this is implemented in the latest MNE versions, along with cross-validation to select the optimal covariance matrix estimation method (and regularization parameters) for each particular dataset [5,7]. All three options have strong supporters and critics amongst experimenters and reviewers, and arguments such as “magnetometers can detect deeper sources”, “gradiometers are less noisy”, or “using magnetometers only (gradiometers only) means discarding 2/3 (1/3) of the data” are often heard. However, are these arguments valid after SSS? In this work, we address this question and argue that, after (t)SSS, magnetometers and gradiometers contain the same information. To tackle this issue, we provide theoretical arguments and compare experimentally resting source reconstructions and sensor and source space task-related activity estimated from magnetometers and gradiometers, with and without SSS. 

## 2. Materials and Methods

### 2.1. Theoretical Reasoning

The initial assumption in SSS is the presence of three separate volumes: (a) an inner volume containing the inside (brain) currents $j_{in}$, (b) an intermediate volume that includes the MEG sensors and is current-free, and (c) an outer volume containing the external currents $j_{out}$. The magnetic field B(r) at a source-free sensor position r is the superposition of the fields created by $j_{in}$ and $j_{out}$:

(1) $$B\left(r\right)=B_{in}\left(r\right)+B_{out}\left(r\right).$$

As proven in [1,12], under the quasistatic approximation, $B_{in}\left(r\right)$ and $B_{out}\left(r\right)$ can be written as separate series expansions:

(2) $$B_{in}\left(r\right)=\sum_{l=1}^{\infty }\sum_{m=-l}^{l}\alpha_{lm}F_{l}\left(r\right),$$

(3) $$B_{out}\left(r\right)=\sum_{l=1}^{\infty }\sum_{m=-l}^{l}\beta_{lm}G_{l}\left(r\right),$$

where $F_{l}\left(r\right)$ and $G_{l}\left(r\right)$ are functions derived from spherical harmonics and scale with $1/r^{l+2}$ and $r^{l}$, respectively. For the sake of brevity, their analytic formulation is not written here, but can be found elsewhere [1]. $\alpha_{lm}$ and $\beta_{lm}$ are coefficients that depend on the current distributions $j_{in}$ and $j_{out}$, and are independent of the target position ***r***.

$B_{in}$ and $B_{out}$ at each of the MEG coils position $r_{1},\ldots ,\;r_{Ncoils}$ can then be expressed as a linear combination of $\alpha_{lm}$ and $\beta_{lm}$, after truncating (2) and (3) to $l\leq L_{in}$ and $l\leq L_{out}$, respectively, and computing $F_{l}\left(r\right)$ and $G_{l}\left(r\right)$ at $r_{1},\ldots ,\;r_{Ncoils}$. The series expansions are usually truncated to $L_{in}=8$ and $L_{out}=3$, since they are considered to produce a negligible residual [1,3]. This would yield $n_{in}={\left(L_{in}+1\right)}^{2}-1=80$ inside terms and $n_{out}={\left(L_{out}+1\right)}^{2}-1=15$ outside terms. It is important to note that the number of inside components ($n_{in}=80$) is largely inferior to the number of magnetometers (102) or gradiometers (204), and roughly represents the dimensionality of the MEG spatial information (for each time point separately). Although this number could seem low at first sight, it can be understood when examining the spatial decay of magnetic fields generated by dipolar brain sources. We refer the reader to [13] for a deeper discussion on the topic. As an illustrative example, the authors concluded that an MEG system with 61 detector elements (each comprising two planar gradiometers) is well above the critical sensor density that ensures no spatial aliasing, provided that the distance from the sensors to the to the closest cortical source is at least 35 mm. 

Then, the magnetic field (normal component) at each of the coils’ positions can be written as:

(4) $$B_{coil}\approx \left[T_{in,coil}\;T_{out,coil}\right]\left[\begin{array}{c} x_{in}\\ x_{out} \end{array}\right],$$

where $T_{in,coil}$ and $\;T_{out,coil}$ are $N_{coils}\times n_{in}$ and $N_{coils}\times n_{out}$ matrices, respectively, which are computed from the system’s geometry based on $F_{l}\left(r\right)$ and $G_{l}\left(r\right)$; and $x_{in}$ and $x_{out}$ are vectors of length $n_{in}$ and $n_{out}$ that contain the $\alpha_{lm}$ and $\beta_{lm}$ coefficients.

The MEG measurement at each sensor s is $M_{s}=B_{coil,x}$ for magnetometers (where x is the index of the single pick up coil in magnetometer s) and $M_{s}=B_{coil,\;x}-B_{coil,\;y}$ for gradiometers (where x and y are the indices of the two pick-up coils in gradiometer s). The vector of MEG measurements ***M*** can be then expressed as:

(5) $$M\approx Sx=\left[S_{in}\;S_{out}\right]\left[\begin{array}{c} x_{in}\\ x_{out} \end{array}\right],$$

where the rows of $S_{in}$ ($S_{out}$) are rows of $T_{in,coil}$ ($T_{out,coil}$) for magnetometers and are the subtraction of two $T_{in,coil}$ ($T_{out,coil}$**)** rows for gradiometers.

Finally, the inside and outside components are estimated from $\hat{x}=\left[\begin{array}{c} x_{in}\\ x_{out} \end{array}\right]=S^{-1}M$, and a cleaner version of the MEG measurements is estimated by projecting the inside components only:

(6) $$\hat{M_{in}}\approx S_{in}{\hat{x}}_{in}.$$

In summary, a single set of $n_{in}$ inside components $x_{in}$ (usually $n_{in}\leq 80)$ is estimated using all channels, magnetometers and gradiometers (bad channels should of course be discarded). These $n_{in}$ inside components are then projected into all 306 channels (102 magnetometers and 204 gradiometers). 

### 2.2. Experimental Source Reconstructions from Magnetometers and Gradiometers

To further explore the relation between magnetometers and gradiometers after SSS, in this section we compare source reconstructions estimated separately from both sensor types in real MEG recordings.

#### MEG Acquisition and Source Estimation

The resting state data used here was recorded for a test-retest reliability project. Details on data acquisition, preprocessing, and source reconstruction can be found in [14]. Briefly, 4 min resting state eyes closed data from 16 healthy subjects (age 30.4 ± 5.8, ten female) were employed here. MEG recordings were acquired with an Elekta Neuromag^®^ Vectorview system with 306 sensors (102 magnetometers and 204 planar gradiometers), inside a Vacuumschmelze^®^ magnetically shielded room. Subjects’ heads were digitized with a Fastrak Polhemus, and four coils were attached to the forehead and mastoids so that the head position on the MEG helmet was continuously determined. Activity in electrooculogram channels was also recorded to keep track of ocular artifacts. Signals were sampled at 1000 Hz with an online filter of bandwidth 0.1–300 Hz. 

SSS and tSSS were applied to the raw resting state data with Maxfilter (Version 2.2) and its default parameters ($L_{in}$ = 8, $L_{out}$ = 3, tSSS correlation window = 10 s, and tSSS correlation limit = 0.9). We note that, although the data presented in subsequent sections corresponds to the SSS-filtered dataset, we obtained similar results with tSSS. Bad channels were visually detected, and were not included in the SSS/tSSS estimation. Jump, muscle and ocular artefacts were detected using FieldTrip [15], and non-overlapping artefact-free 6-s epochs were located. Data was bandpass filtered in [2–10] Hz with a finite impulse response (FIR) filter of order 1000.

Source and forward models were built individually after segmenting each subject’s T1-weighted MRI with Freesurfer (Version 5.1.0), [16,17], downsampling and realigning surfaces, and estimating leadfield matrices with MNE software [7]. Linearly constrained minimum variance (LCMV) beamformer [18] was used to perform source reconstruction. For each subject and source i, we computed beamformer filters as:

(7) $$W_{i}={\left[{L_{i}}^{T}C_{inv}L_{i}\right]}^{-1}{L_{i}}^{T}C_{inv},$$

where $L_{i}$ is the $N_{sensors}\times 3$ leadfield matrix between each sensor and the i-th source for three brain current orientations (along Cartesian axes x, y and z). C is the $N_{sensors}\times N_{sensors}$ sensor covariance matrix and is estimated using all samples in the resting state clean trials. $C_{inv}$ is an estimate of the inverse of C:

(8) $$C_{inv}=pinv\left(C+\lambda \frac{trace\left(C\right)}{N_{sensors}}I\right),$$

where I is the identity matrix and λ > 0 is called the regularization factor. λ is a dimensionless magnitude and represents the fraction of $\frac{trace\left(C\right)}{N_{sensors}}$, which is added to the diagonal of C in order to render it more robust to matrix inversion. This is a common convention in the literature [19], and it is used in the popular FieldTrip toolbox [15]. Regularizing is equivalent to adding uncorrelated noise to the sensor measurements, but it is necessary for the stability of the inversion of the covariance matrix C. This is especially crucial after SSS, since SSS projects back only 60–80 inside coefficients, and yields rank-deficient covariance matrices. The robustness of the $C_{inv}$ matrix inversion can be quantified through:

(9) $$cn=\mathrm{cond}\left(C+\lambda \frac{trace\left(C\right)}{N_{sensors}}I\right)$$

where cond refers to the 1-norm condition number, a dimensionless measure defined as $\mathrm{cond}\left(A\right)=||A||\cdot ||A^{-1}||$ [20]. Matrices with condition numbers close to one are well conditioned with respect to inversion. Conversely, matrices with higher condition numbers are ill conditioned with respect to inversion; small changes in the original matrix A can lead to big changes in the estimate of the inverse matrix $A^{-1}$. 

For each source location i, the orientation $\eta_{i}$ (1 × 3 row vector) of the source activity was determined as the one maximizing the source power ${W_{i}}^{T}Cw_{i}$, and the beamforming filter was projected into this direction: $W_{i,\eta }=\eta_{i}W_{i}$. Finally, we derived source time series as:

(10) $$s_{i}\left(t\right)=W_{i,\eta }M\left(t\right)$$

For each subject, source time series were extracted for magnetometers and gradiometers separately for varying regularization parameters λ_mag_ and λ_grad_. The similarity between magnetometer and gradiometer source reconstructions was evaluated with the Pearson correlation between reconstructed source time series. The effect of the magnetometers vs. gradiometer choice on resting state power spectrum and functional connectivity estimates was evaluated with intraclass correlation coefficient (ICC type 1-1, following [21]).

## 3. Results

### 3.1. Correlation between Magnetometer and Gradiometer Source Reconstructions after SSS, as a Function of the Regularization Factor λ

**Figure 1:**
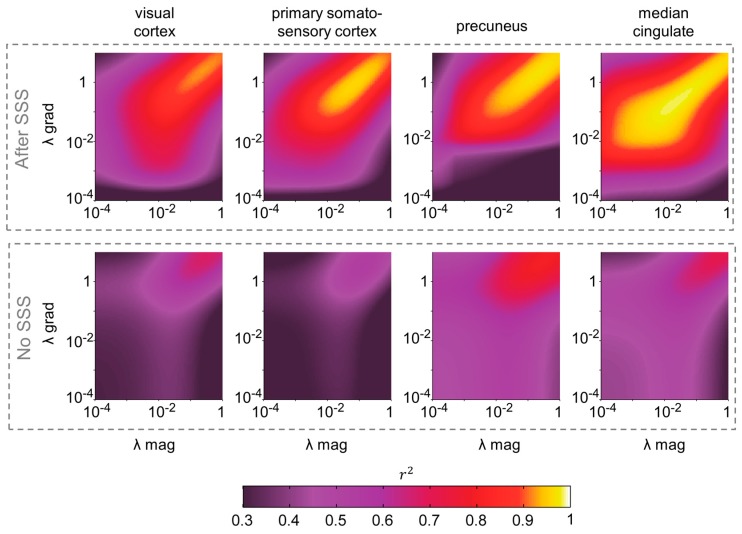
Correlation between source time series derived from magnetometers and gradiometers with and without SSS. Squared Pearson correlation coefficients averaged across subjects are shown for four selected sources as a function of the regularization parameters λ for magnetometer (*x*-axis) and gradiometer (*y*-axis) beamforming reconstructions. The selected sources were located in MNI space: visual cortex (MNI: [−41, −77, 3] mm), primary somatosensory cortex (MNI: [−38, −27, 52] mm), precuneus (MNI: [1, −57, 28] mm) and median cingulate (MNI: [−2, 12, 40] mm).

For each subject, we computed source reconstruction separately for magnetometers and gradiometers, with and without SSS, for 80 regularization factors between λ = 10^−4^ and λ = 1. For brevity and simplicity, we focused here on four sources of interest, which are spread across the cortex and commonly are used in the neuroimaging literature: visual cortex (MNI coordinates [−41, −77, 3] mm), primary somatosensory cortex (MNI: [−38, −27, 52] mm), precuneus (MNI: [1, −57, 28] mm) and median cingulate (MNI: [−2, 12, 40] mm). Squared Pearson correlation coefficients r^2^ were computed between source time series derived from magnetometers and gradiometers for each pair of λ_mag_ and λ_grad_ and averaged over trials and subjects (see Figure 1). Without SSS, r^2^ values (squared Pearson correlation coefficients) were moderate, ranging between 0.3 and 0.5, and reaching their highest values (0.6–0.8) for high regularizations approaching λ = 1. However, they were much higher for SSS-filtered data, reaching values of r^2^ > 0.9 for λ_mag_ and λ_grad_ > 0.01 (*p* < 10^−4^ for all combinations λ_mag_ and λ_grad_ > 0.01, paired samples *t*-test comparing r^2^ values with and without SSS). The λ_mag_ and λ_grad_ reaching the highest values of r^2^ > 0.9 were positively related, in a seemingly log-log dependence. Of note, equivalent results were obtained when comparing the source reconstructions using each type of sensor separately with that obtained with the whole magnetometer + gradiometer dataset, normalizing the variance of both sensor types as in [22]. Results can be found in Supplementary Figure S1.

Notably, the change in correlation strength before and after SSS is not exclusively due to an increase in the similarity between magnetometers and gradiometers introduced by the SSS backprojection. In fact, MEG data before SSS is affected by a considerable external interference, which can influence both sensor types differently. In particular, we could expect that, before SSS, magnetometer data contained a higher noise level than gradiometer data, therefore producing noisier source estimates and decreased correlation between derived source time series. We can use the ratio of the maximum sensor space magnetometer amplitude before and after SSS as a rough estimate of the intensity of noise that is eliminated during SSS. We found that this noise estimate correlated negatively with the correlation strength between magnetometer- and gradiometer-derived source time series before SSS (*p*-value = 0.027). In other words, the greater the noise level in the raw recordings without SSS, the smaller the similarity between magnetometer and gradiometer source reconstructions. More details can be found in Supplementary Figure S2.

**Figure 2:**
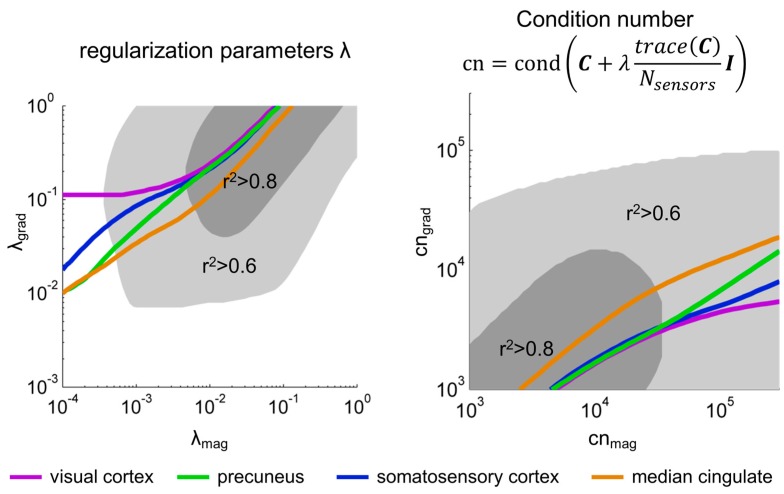
The correlation between magnetometer and gradiometer source reconstruction after SSS depends on the regularization parameter λ and the condition number cn of the sensor covariance matrix. In the left, the lines display, for each λ_mag_, λ_grad,max_—the λ_grad_ that yields maximum subject average correlation r^2^ between magnetometer and gradiometer source reconstructions. The gray surfaces span the area for which the subject average r^2^ > 0.6 (lighter gray) and r^2^ > 0.8 (darker gray) for all five sources considered. In the right side, an equivalent plot is built with axes corresponding to the condition numbers of the regularized magnetometers and gradiometers covariance matrices (cn_mag_ and cn_grad_, respectively).

We further explored this dependence by selecting, for each source and *λ*_mag_ separately, the *λ*_grad_ for which the highest *r^2^* was obtained. As shown in Figure 2, the relation between λ_mag_ and *λ*_grad,max_ was indeed monotonically increasing in a rather linear fashion for *λ*_mag_ > 0.01. A least squares linear fit $\log_{10}\left(\lambda_{\mathrm{grad},\max}\right)$ = $a\cdot \log_{10}\left(\lambda_{\mathrm{mag}}\right)+b$ was computed for each subject separately, averaging over the four sources of interest, resulting in a = 0.56–0.71, b = 0.59–0.80 (95% confidence intervals, *t*-test across subjects). For all subjects, the proportion of explained variance of this linear model was between 0.86 and 0.999. This means that equivalent source reconstructions are obtained for λ_grad_ > λ_mag_. For instance, for a λ_mag_ = 0.01, the corresponding λ_grad_ yielding the most similar source reconstructions is λ_grad_ = 0.29. When focusing on the condition numbers of $C+\lambda \frac{trace\left(C\right)}{N_{sensors}}I$, instead of the regularization factors λ, we also observed a rather linear and positive relation between cn_mag_ and cn_grad,max_. The minimum square linear fits $\log_{10}\left({\mathrm{cn}}_{\mathrm{grad},\max}\right)$ = $a\cdot \log_{10}\left({\mathrm{cn}}_{\mathrm{mag}}\right)+b$ for each subject resulted in a = 0.51–0.66, b = 0.52–1.15, proportion of explained variance 0.90–0.99 (95% confidence intervals). Equivalent source reconstructions are obtained for cn_grad_ < cn_mag_.

### 3.2. Spatial Dependence of the Correlation between Magnetometer and Gradiometer Source Reconstructions

**Figure 3:**
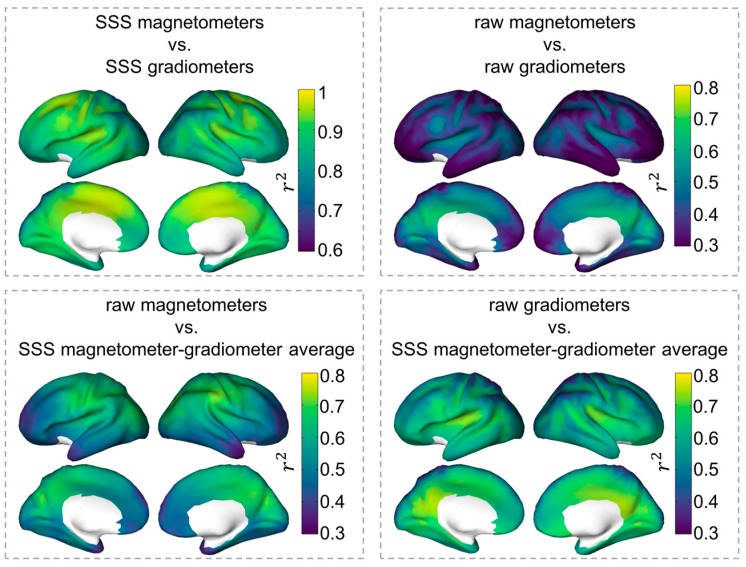
Correlation between source time series for λ_mag_ = 0.01 and λ_grad_ = 0.29. The brain surfaces display the subject-averaged squared Pearson correlation coefficients between pairs of source time series estimated with different sensor types and with/without SSS. Note that the limits of the color bars differ across panels.

To evaluate the whole-brain spatial relationship between magnetometer and gradiometer source reconstructions, we separately computed the correlations between source time series extracted with both sensor types using λ_mag_ = 0.01 and λ_grad_ = 0.29, respectively, for all cortical sources. Figure 3 shows the spatial distribution of the average r^2^ between magnetometer and gradiometer source reconstructions across subjects, using both the raw and the SSS-filtered datasets. After SSS, r^2^ > 0.8 across the brain. This correlation was much weaker for the raw dataset without SSS, with r^2^ < 0.4 for most cortical regions and r^2^ > 0.5 for posterior and deeper regions around visual cortex, posterior cingulate and precuneus. When comparing source reconstructions with and without SSS, the former were more strongly correlated (r^2^ > 0.6), with raw magnetometer source reconstructions in posterior central, parietal, and posterior regions, and with raw gradiometer source reconstructions in frontal, temporal and cingulate regions.

### 3.3. Inter-Pipeline Reliability of Power and Functional Connectivity Values: Impact on the Choice of Magnetometers or Gradiometers

One may argue that the aforementioned results only apply to the reconstructed source time series in the time domain, whereas the spectral properties or the functional connectivity (FC) patterns obtained from both types of sensors may differ. To estimate the impact that the choice of magnetometers or gradiometers would have in the outcome of the spectral properties and FC in a real experiment, we quantified the difference between the magnetometer and gradiometer-derived values of these target measures. Power spectra were calculated from source time series with the multitaper method using Hamming windows and 1 Hz smoothing and normalized to the overall power in the [2–30] Hz. We estimated relative power for the following frequency bands: delta ([2–4] Hz), theta ([4–8] Hz), alpha ([8–13] Hz) and beta ([13–30] Hz), and averaged over the 66 cortical regions of the Desikan-Killiany atlas [23]. As an estimate of FC, we computed the phase-locking value (PLV) between all pairs of regions in the delta, theta, alpha, and beta bands. More details on the implementation of power and PLV estimation can be found in [14,24].

**Figure 4:**
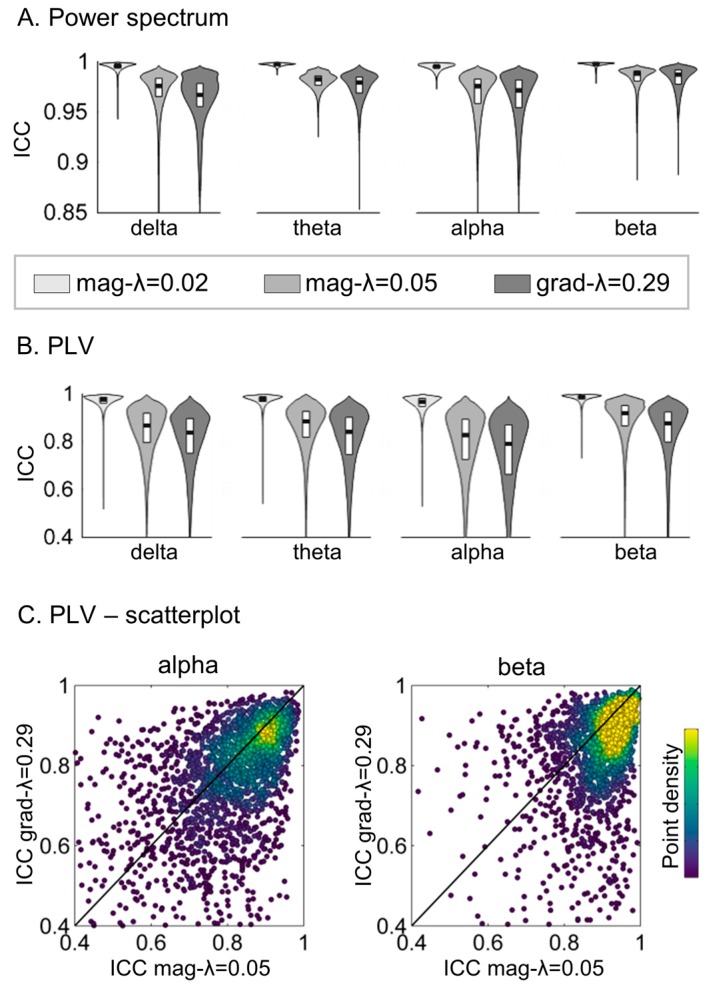
Inter-pipeline reliability of power spectrum and phase locking value (PLV) estimates. The reliability was evaluated with the ICC (Intraclass correlation coefficient), using as a reference the mag-λ = 0.01 pipeline (choosing magnetometers for source reconstruction and a regularization factor of λ = 0.01). We compared three pipelines against this reference: mag-λ = 0.02 (using magnetometers and λ = 0.02), mag-λ = 0.05 (using magnetometers and λ = 0.05) and grad-λ = 0.29 (using gradiometers and λ = 0.29). (**A**) Violin plots of the distribution of ICC coefficients for relative power estimates in the delta, alpha, beta and gamma bands across the 66 cortical regions of the Desikan-Killiany atlas. (**B**) Violin plots of the distribution of ICC for PLV across pairs of regions (or links) for each frequency band. (**C**) Scatterplot of the ICC values obtained with the mag-λ = 0.05 and the grad-λ = 0.29 pipelines. Each dot represents a single connection, and the color displays the density of points.

The inter-analysis pipeline reliability of the power and PLV estimates was quantified with the intraclass correlation coefficient (ICC type 1-1, following [21]), comparing power and PLV values obtained with magnetometers and regularization mag-λ = 0.01 and gradiometers with regularization grad-λ = 0.29. Figure 4 shows the distribution of ICC values across regions and links. For comparison, we also computed the reliability between the analysis pipelines using magnetometers only, but with different λ coefficients (always keeping the dataset obtained with magnetometers with λ = 0.01 as a reference), and these are included in Figure 4. We found excellent inter-pipeline reliability (ICC > 0.85) for the power spectra for all analysis pipeline combinations. ICC values for PLV were however smaller. Although for more than 3/4 of the links ICC > 0.8, which is usually regarded as an excellent reliability [25], the ICC values spanned a broader interval ([0.4–1]) when comparing mag-λ = 0.01 and grad-λ = 0.29. This was, however, also the case when comparing within a single sensor type mag-λ = 0.01 and mag-λ = 0.05. To further evaluate whether the impact on reliability was driven by the regularization or by the sensor choice, the dependence between ICC values obtained with grad-λ = 0.29 and mag-λ = 0.05 (keeping mag-λ = 0.01 as a reference) was explored (Figure 4C). Both magnitudes were strongly correlated (Pearson r^2^ = 0.13–0.26, *p*-value < 10^−10^). This means that the links with smaller ICC in grad-λ = 0.29 tend to be also the links with smaller ICC in mag-λ = 0.05. This fact could indicate that the regularization intensity rather than the sensor choice causes the drop on ICC. 

### 3.4. Generalizability of the Previous Results

In order to demonstrate the reproducibility and generalizability of our results, we undertook similar magnetometer and gradiometer comparisons in an external dataset, recorded in a different MEG site and with a different protocol. We used MEG recordings during a passive visual task for 37 healthy volunteers (between 18 and 29 years age, mean 24.1). Data were obtained from the CamCAN repository (available at http://www.mrc-cbu.cam.ac.uk/datasets/camcan/) [26,27]. tSSS was applied with Maxfilter (version 2.2) $L_{in}$ = 8, $L_{out}$ = 3, tSSS correlation window = 10 s, and tSSS correlation limit = 0.98. A detailed description of the methods used in this section can be found in the Supplementary Material.

**Figure 5:**
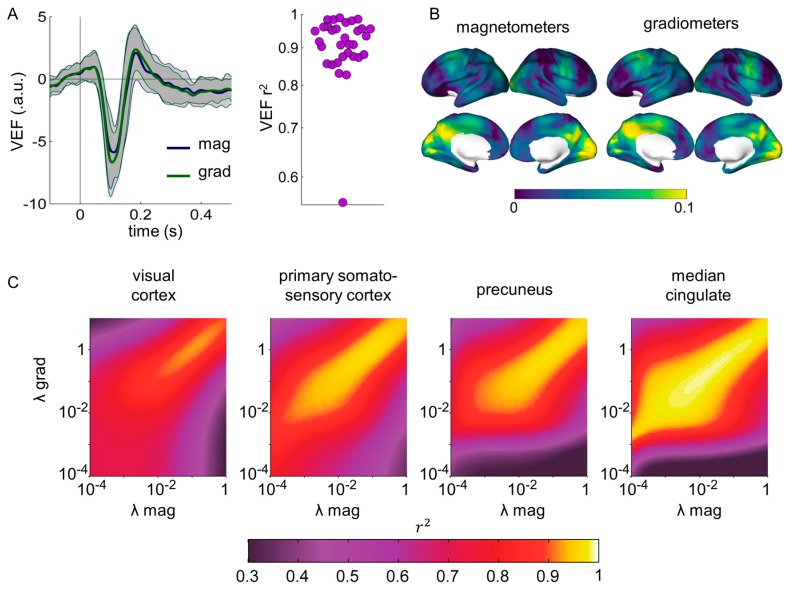
Similarity of the evoked and ongoing activity during a passive visual task estimated with magnetometers and gradiometers after SSS. (**A**) Sensor space Visual Evoked Fields (VEF). The gray areas represent the standard deviation across subjects. The squared Pearson correlation coefficients (r^2^) between the magnetometer and gradiometer-derived VEFs are displayed for each subject. (**B**) Source activation at 60–160 ms after stimulus presentation compared to baseline (−100–0 ms). (**C**) Correlation between source time series derived from magnetometers and gradiometers. Beamforming filters and source power values were estimated from the average covariance matrix across all trials (from −0.1 to 0.9 s relative to stimulus onset).

We first focused on the Visual Evoked Fields (VEFs). For each subject and sensor type, a representative VEF was computed directly from sensor data using a principal component approach, similar to that in [28]. Results are shown in Figure 5A. Magnetometer- and gradiometer-derived sensor space VEF were extremely correlated, with r^2^ > 0.9 for all but one subject. Notably, magnetometer- and gradiometer-derived source space VEFs in the left and right primary visual cortices (MNI coordinates [−11, −81, 7] mm and [11, −78, 9] mm, respectively) were also strongly correlated at the subject level with similar results for both visual cortices. Maps of source power in the 60–160 ms interval compared to baseline (−100 to 0 ms) were computed from magnetometer and gradiometer data separately, leading to similar source activation plots of around 10% power increase in posterior regions (Figure 5B).

Additionally, we replicated the analyses performed in Section 3.1 in the passive visual task, focusing on the ongoing activity during the whole task (−100 to 900 ms relative to stimulus onset). Similarly to Section 3.1, source reconstructions were performed separately for magnetometers and gradiometers for regularization factors between λ = 10^−4^ and λ = 1, focusing on four representative sources. Squared Pearson correlation coefficients r^2^ were computed between source time series derived from magnetometers and gradiometers for each pair of λ_mag_ and λ_grad_ and averaged over trials and subjects (Figure 5C). The same patterns than in Section 3.1 were recovered, reaching correlations of r^2^ > 0.9. 

## 4. Discussion

In this work, we have demonstrated both theoretically and experimentally that magnetometer and gradiometer data after SSS contain equivalent information, and therefore produce very similar sensor-space VEFs and source space resting state activity estimates and task-related activity maps (r^2^ > 0.8). Although these results may not come as a surprise for most Elekta users experienced with SSS and source reconstruction, there is substantial controversy in the general neuroimaging community on the selection of magnetometers and gradiometers. The present work provides a focused analysis on this issue, which we hope will guide MEG users who are designing analysis plans, contribute to avoiding additional lengthy discussions during scientific peer-review, and inform researchers who are doubtful about the different source reconstruction approaches.

Currently, the community using Elekta MEG devices is divided among different options: using magnetometers only [29], gradiometers only [30], both sensor types simultaneously [31,32], or both sensor types separately across main manuscript and supplementary information [24]. In this work, we have demonstrated that all these approaches can be regarded as equivalent when using SSS for MEG data denoising. We note, however, that the choice of magnetometers and gradiometers is relevant when working in sensor space, since both sensor types yield different topographies for the same source activation. While magnetometers have a circular sensitivity distribution, planar gradiometers have maximum sensitivity directly under the sensors [9]. Furthermore, planar gradiometers can be combined to produce sensor-space pseudo current maps, which provide a more accurate estimation of the underlying current distribution than magnetometer-derived topographies [33]. 

A crucial factor in ensuring comparability between magnetometer and gradiometer source reconstructions is an appropriate regularization of the covariance matrices. The importance of regularization in MEG/EEG beamforming has been established previously [34,35,36]. Brookes et al. [37] demonstrated that shorter experiment times, smaller frequency bandwidths and increasing numbers of sensors produce a higher error in the covariance matrix estimation, and therefore require greater regularization, which comes at the expense of a loss in spatial resolution. After (t)SSS, the effective dimensionality of magnetometer and gradiometer covariance matrices is equal to the number of inside SSS components, which is typically in the range 60–80. Since Elekta MEG devices contain twice as many gradiometers as magnetometers, after SSS the gradiometer covariance matrix has a higher condition number (is less invertible) than the magnetometer covariance matrix. When using the popular approach of defining the regularization parameter λ as the fraction of the average diagonal of the covariance matrix, which is added to the covariance matrix’s diagonal before inversion [15,19], the highest agreement between magnetometer- and gradiometer-derived source time series was obtained for λ_grad_ > λ_mag_. Notably, other methods for covariance matrix estimation and regularization have been introduced [11], and they may yield a different dependence between the similarity of magnetometer- and gradiometer-derived source reconstructions and regularization strength. This dependence could also be altered when using inverse models other than beamforming. In fact, beamformers were found to be more strongly affected than minimum norm estimates by errors in covariance matrix estimation [38]. 

Although selecting higher regularizations for gradiometers than for magnetometers yields comparable condition numbers and the highest level of correlation between the source time series, having different regularization intensities (and therefore the different intensity of added noise) can affect the spatial properties of magnetometers and gradiometers. To explore this, we evaluated the inter-pipeline reliability of conventional MEG measures (power and functional connectivity) obtained with magnetometers with λ = 0.01 and gradiometers with λ = 0.29 (combination yielding source time series correlation r^2^ > 0.8). Power values estimated with both analysis pipelines were remarkably similar (ICC > 0.85). Functional connectivity values were more variable; although for most links and frequency bands ICC > 0.8, ICC values in the 0.4–0.8 range were also obtained. ICC comparing pipelines using magnetometers only and different values of λ were similarly affected, indicating that the regularization is causing the difference in FC estimates obtained with magnetometers and gradiometers. Such a result is not particularly surprising, since the effect of regularization on the spatial resolution of source reconstruction is well known [34,35,36].

## 5. Conclusions

We have highlighted here an often-overlooked fact: magnetometer and gradiometer data after SSS are derived from the same set of SSS inside components (usually a maximum of 80 components), which represent the most dominating magnetic field patterns of interest in any multichannel MEG measurement. We have then demonstrated that, after SSS, magnetometer and gradiometer datasets produce very similar outcome measures: resting state power spectral and functional connectivity estimates (average ICCs of 0.8–0.98) and visual evoked fields (r^2^ > 0.9). These results unify different analysis pipelines existing in the literature, and prove that the choice of magnetometers or gradiometers in source reconstruction after SSS has a small impact on the outcomes of MEG studies. In fact, other analysis parameters such as the beamforming regularization strength could have a stronger impact than the sensor type choice. These findings support the relevance of developing of new source analysis methods that could work directly in the SSS space, and thereby avoid projecting SSS inside components back into sensor space and using strong regularization to account for badly conditioned covariance matrices. 

## Supplementary Materials

Supplementary figures and methds can be found online at http://www.mdpi.com/1424-8220/17/12/2926/s1. Figure S1. Correlation between source time series derived from magnetometers only and magnetometers + gradiometers combined, depending of the λ regularization parameter. Squared Pearson correlation coefficients averaged across subjects are shown for four selected sources, as a function of the regularization parameters λ for magnetometer (*x*-axis) and magnetometer + gradiometer (*y*-axis) beamforming reconstructions. For the (mag + grad) dataset, data for both sensor types were variance normalised. Figure S2. Dependence between the squared Pearson correlation coefficient r^2^ between source time series derived from magnetometers and gradiometers and the noise estimate in the raw recordings (*z_noise_*). Each point represent a single recording (subject). r^2^_max,raw_ and r^2^_max_, SSS is the strongest r^2^ across pairs of regularization parameters λ_mag_ and λ_grad_ for datasets without and with SSS, respectively, for each subject separately (averaging r^2^ maps across the four sources of interest). *z_noise_*) is defined as the ratio between the maximum magnetometer amplitude (across channels and time) before SSS and after SSS and is computed for each subject and trial separately, and then averaged over trials.
