# Supplementary material for: Choice of Magnetometers and Gradiometers after Signal Space Separation
Source: Sensors (Basel). 2017 Dec 16;17(12):2926. doi: 10.3390/s17122926 (PMC5751446; doi:10.3390/s17122926)
Supplement: Supplementary file 1 [file sensors-17-02926-s001.pdf]

Supplementary Material to: *Choice of magnetometers and gradiometers after signal space separation.* Sensors

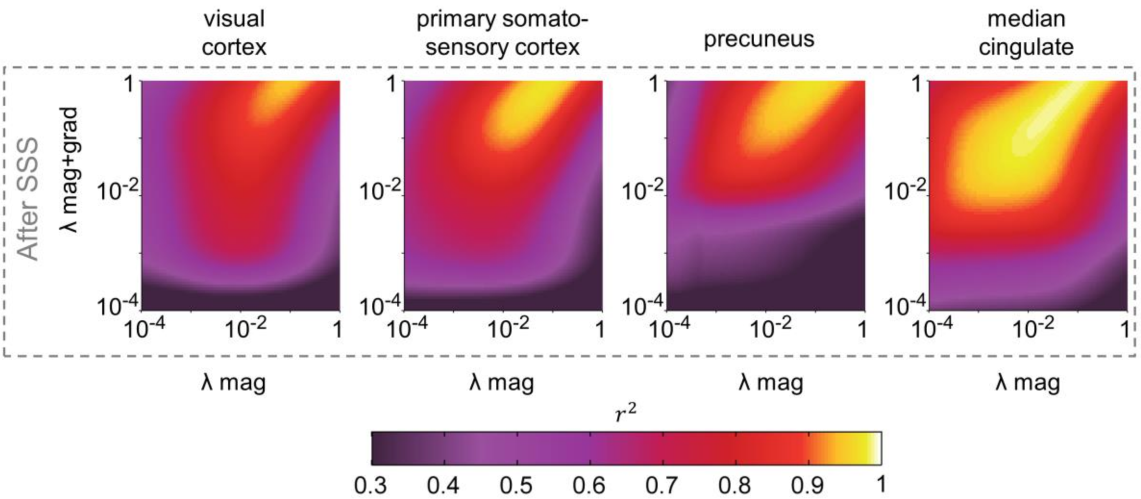

Figure S1. Correlation between source time series derived from magnetometers only and magnetometers+gradiometers combined, depending of the λ regularization parameter. Squared Pearson correlation coefficients averaged across subjects are shown for four selected sources, as a function of the regularization parameters λ for magnetometer (x-axis) and magnetometer+gradiometer (y-axis) beamforming reconstructions. For the (mag+grad) dataset, data for both sensor types were variance normalised.

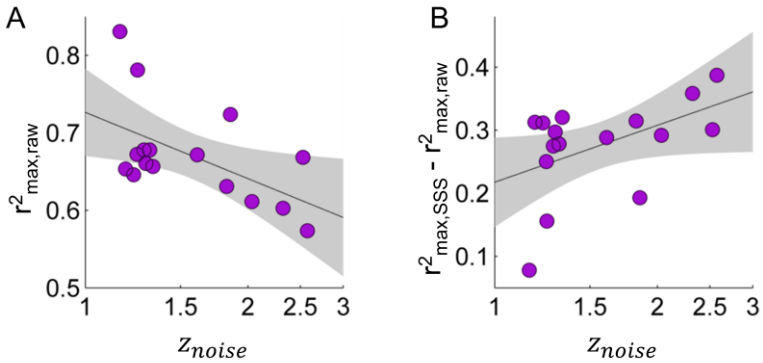

Figure S2. Dependence between the squared Pearson correlation coefficient r^2 between source time series derived from magnetometers and gradiometers and the noise estimate in the raw recordings (z_{noise}). Each point represent a single recording (subject). $r^2_{max,raw}$ and $r^2_{max,SSS}$ is the strongest r^2 across pairs of regularization parameters λ_{mag} and λ_{grad} for datasets without and with SSS, respectively, for each subject separately (averaging r^2 maps across the four sources of interest). z_{noise} is defined as the ratio between the maximum magnetometer amplitude (across channels and time) before SSS and after SSS and is computed for each subject and trial separately, and then averaged over trials.

Supplementary Methods:

The visual stimulation task employed here is part of the Cam-CAN database acquired using a 306-channel Vectorview system (Elekta Neuromag, Helsinki). Data was acquired with 1000 Hz sampling rate and an online band-pass filter between 0.03 and 330 Hz was applied. MEG recordings took place during passive audio-visual sensory task, with 120 trials of unimodal stimuli presented every 1 s. In half of the trials participants listened to an auditory tone presented for 300 ms, and in the other half they watched two circular checkerboards to the left and right of a fixation cross for 34 ms with no response required from the subject. Our analyses focused on trials involving visual stimuli. A detailed description of the exact task settings and session protocol can be found elsewhere [1–3].

A standard preprocessing algorithm was used: raw MEG data were filtered with tSSS using the settings described in CamCAN dataset (http://www.mrc-cbu.cam.ac.uk/datasets/camcan/). Bad channels were detected automatically with Maxfilter, applying tSSS with a correlation window of 10s and a correlation limit of 0.98. Artifacted segments were detected using FieldTrip and visually confirmed afterwards. MEG signals were corrected for ocular artifacts using Independent Component Analysis (ICA). MEG data were filtered into [2-35] Hz and segmented into trials (-100ms to 900ms relative to visual stimulus onset). Only non-artifacted trials were kept for further analyses (number of trials: mean: 57. 5, SD: 6.1).

Sensor Space ERP: We followed a similar approach than [2]. ERF were defined as the first Principal Component of the Nchannels x Ntimesamples sensor space ERF matrix over the -0.1–0.5s interval, relative to stimulus onset. For the sensor space analysis, only subjects for which the first Principal Component explained over 40% of the variance of the sensor space ERF matrix were included (32/37 subjects).

Source space analysis: Forward models were solved using 3-shell Boundary Element Method. Source positions were defined in MNI space in a regular grid with 1 cm spacing. The inverse problem was solved with beamforming, following equation (10).

References

1. Taylor, J. R.; Williams, N.; Cusack, R.; Auer, T.; Shafto, M. A.; Dixon, M.; Tyler, L. K.; Cam-CAN; Henson, R. N. The Cambridge Centre for Ageing and Neuroscience (Cam-CAN) data repository: Structural and functional MRI, MEG, and cognitive data from a cross-sectional adult lifespan sample. *Neuroimage* **2017**, *144*, 262–269, doi:10.1016/j.neuroimage.2015.09.018.
2. Price, D.; Tyler, L. K.; Neto Henriques, R.; Campbell, K. L.; Williams, N.; Treder, M. S.; Taylor, J. R.; Brayne, C.; Bullmore, E. T.; Calder, A. C.; Cusack, R.; Dalgleish, T.; Duncan, J.; Matthews, F. E.; Marslen-Wilson, W. D.; Rowe, J. B.; Shafto, M. A.; Cheung, T.; Davis, S.; Geerligs, L.; Kievit, R.; McCarrey, A.; Mustafa, A.; Samu, D.; Tsvetanov, K. A.; van Belle, J.; Bates, L.; Emery, T.; Erzinglioglu, S.; Gadie, A.; Gerbase, S.; Georgieva, S.; Hanley, C.; Parkin, B.; Troy, D.; Auer, T.; Correia, M.; Gao, L.; Green, E.; Allen, J.; Amery, G.; Amunts, L.; Barcroft, A.; Castle, A.; Dias, C.; Dowrick, J.; Fair, M.; Fisher, H.; Goulding, A.; Grewal, A.; Hale, G.; Hilton, A.; Johnson, F.; Johnston, P.; Kavanagh-Williamson, T.; Kwasniewska, M.; McMinn, A.; Norman, K.; Penrose, J.; Roby, F.; Rowland, D.; Sargeant, J.; Squire, M.; Stevens, B.; Stoddart, A.; Stone, C.; Thompson, T.; Yazlik, O.; Barnes, D.; Dixon, M.; Hillman, J.; Mitchell, J.; Villis, L.; Henson, R. N. A. Age-related delay in visual and auditory evoked responses is mediated by white- and grey-matter differences. *Nat. Commun.* **2017**, *8*, 15671, doi:10.1038/ncomms15671.
3. Shafto, M. A.; Tyler, L. K.; Dixon, M.; Taylor, J. R.; Rowe, J. B.; Cusack, R.; Calder, A. J.; Marslen-Wilson, W. D.; Duncan, J.; Dalgleish, T.; Henson, R. N.; Brayne, C.; Matthews, F. E. The Cambridge Centre for Ageing and Neuroscience (Cam-CAN) study protocol: a cross-sectional, lifespan, multidisciplinary examination of healthy cognitive ageing. *BMC Neurol.* **2014**, *14*, 204, doi:10.1186/s12883-014-0204-1.
